# Device-based day-to-day and observer variability to quantify dilation capacity in the retinal microcirculation

**DOI:** 10.3389/fphys.2025.1663370

**Published:** 2025-10-15

**Authors:** Lukas Streese, Christoph Hauser, Denis Infanger, Sascha Klee, Dietmar Link, Walthard Vilser, Henner Hanssen

**Affiliations:** ^1^ Department of Sport, Exercise and Health, Medical Faculty, University of Basel, Basel, Switzerland; ^2^ Faculty of Healthcare, Niederrhein University of Applied Sciences, Krefeld, Germany; ^3^ Division Biostatistics and Data Science, Department General Health Studies, Karl Landsteiner University of Health Sciences, Krems an der Donau, Austria; ^4^ Division Optoelectrophysiological Engineering, Department of Computer Science and Automation, Institute of Biomedical Engineering and Informatics, Technische Universität Ilmenau, Ilmenau, Germany

**Keywords:** reproducibility, dynamic retinal vessel analysis, retinal endothelial function, device, clinical implementation

## Abstract

**Introduction:**

Dynamic retinal analysis (DVA) is a validated method to quantify microvascular endothelial function. This study aimed to analyze day-to-day variability, intra- and interobserver variability and differences between two device generations.

**Methods:**

DVA was performed on two separate days and on two devices each, the DVA 2.0 and the DVA 3.0. One reader analyzed 20 signals of maximum arteriolar (aFID) and venular flicker-light induced dilation (vFID) twice to investigate intraobserver variability. A second reader independently analyzed 20 aFID and vFID signals to quantify interobserver variability. The interclass correlation coefficient (ICC) and the 95% confidence interval were used to quantify reliability.

**Results:**

The analysis of 26 participants (mean age 43 ± 14 years) showed moderate to good day-to-day variability for aFID (ICC 0.81 (0.57, 0.92), p = 0.037) and vFID (0.91 (0.80, 0.96), p < 0.001) of DVA 2.0 and low to moderate day-to-day variability for aFID (0.79 (0.49, 0.91), p = 0.076) and vFID (0.87 (0.61, 0.95), p = 0.022) of DVA 3.0. The analyses showed very good intraobserver (aFID and vFID: 0.999 (0.998, 1), p < 0.001) and interobserver variability (aFID: 0.997 (0.993, 0.999), p < 0.001; vFID: 0.998 (0.971, 0.995), p < 0.001). The measurements with devices DVA 2.0 and DVA 3.0 showed a moderate interdevice variability for aFID (0.76 (0.57, 0.89), p = 0.042) and vFID (0.87 (0.74, 0.93), p < 0.001). The ICC of aFID improved for day-to-day variability and interdevice variability after correcting for the baseline diameter.

**Conclusion:**

Consideration of arteriolar baseline diameter variations may further improve day-to-day and interdevice variability. This work underpins the necessity for standardized methods to support clinical implementation of the method and the need to consider arteriolar baseline diameters in future research and clinical applications.

## Introduction

Endothelial dysfunction is a known premorbid condition in the pathophysiology of various cardiovascular (CV) diseases, such as hypertension, atherosclerosis or the development of heart failure (Alexander et al., 2021). Microvascular dysfunction is involved in early pathophysiological processes (Padro et al., 2020). The retinal microcirculation represents a uniquely accessible and sensitive vascular bed to quantify systemic CV risk and monitoring disease progression (Hanssen et al., 2022). A position statement by the European Society of Cardiology has underscored the potential of assessing microvascular function using dynamic retinal vessel analysis (DVA) (Alexander et al., 2020). DVA quantifies maximal flicker light-induced arteriolar (aFID) and venular (vFID) dilation, serving as biomarker of microvascular function (Hanssen et al., 2022). These biomarkers have recently been shown to reflect systemic CV risk (Theuerle et al., 2021; Gunthner et al., 2022) and are able to quantify intervention effects on the microvascular function (Streese et al., 2020; Twerenbold et al., 2023). We recently published normative data for DVA assessments using the Dynamic Vessel Analyzer 2.0 with the ZeissFF450 fundus camera (DVA®; IMEDOS Systems GmbH, Jena, Germany) (Streese et al., 2021a). A new generation device, the Dynamic Vessel Analyzer 3.0 (DVA 3.0) has recently been introduced. There are technical differences between the device versions, particularly regarding the generation of flickering light, which could also have an influence on the vascular response. Version 2.0 uses a halogen lamp (with a red-free filter) as its light source, in which the flickering light is generated by an LCD shutter. Even when closed, this shutter still has a certain amount of residual transmission, which means that only a limited modulation depth can be achieved. Furthermore, the LCD shutter in version 2.0 exhibits a certain amount of inertia, which leads to slower and also different rise and fall times compared to version 3.0. In contrast, the lighting system in version 3.0 is based on light-emitting diodes (LEDs). The flickering light is achieved here by switching the LEDs on and off, which allows for an almost infinite modulation depth. It is not yet clear whether these technical differences could influence the vascular response.

The main aim of this study was to investigate the day-to-day variability of the new DVA 3.0 and the previous generation which was used to define the normative data (DVA 2.0). Further aims were to analyse inter- and intraobserver variability of both devices and the interdevice variability between the DVA 3.0 and DVA 2.0. We hypothesized to find a high reproducibility in both devices and that the differences in absolute values between the devices would be measurable but small and was mostly systematic in nature.

## Methods

### Study design

This single center study was conducted at the Department of Sport, Exercise and Health (DSBG) of the University of Basel. Male and female participants were recruited to undergo measurements of aFID and vFID at two separate time points using both generations of device, the DVA 2.0 and DVA 3.0. The two measurements sessions were scheduled 7 days apart, at the same time of day without any intervention. This study was approved by the Ethics Committee of North-western and Central Switzerland (EKNZ-2021-00086) and was designed and conducted in accordance with the Declaration of Helsinki (World Medical, 2013). All participants signed a written informed consent prior to the first assessment.

### Inclusion and exclusion criteria

Participants aged 18 or older were invited to take part in this study. Recruitment was carried out during an open house event at the DSBG. This open house event addressed the general public in Basel Stadt. Individuals with various social backgrounds took part in this event. Medical history was assessed at the first assessment. Exclusion criteria included untreated CV diseases, any cancer treatments, any acute or chronic eye disease such as macular degeneration, cataract, diabetic retinopathy, glaucoma or any other ocular disease and elevated intraocular pressure (IOP≥ 20 mmHg).

### Retinal microvascular assessments

Pupil dilatation of the right eye was achieved using one drop of tropicamide 0.5% at both time points. Blood pressure was measured after 10 min of rest in a sitting position. DVA was performed in randomized order using both the DVA 2.0 and DVA 3.0 at each time point with a rest period of at least 10 min in between measurements. Device order was randomized using sealed envelopes prepared in advance. An detailed description of the DVA protocol and the analytical procedure can be found elsewhere (Hanssen et al., 2022; Streese et al., 2021a). Briefly, the standard protocol provided by IMEDOS Systems was applied, consisting of three identical cycles with initial 50 s baseline phase, 20 s of flicker light (12.5 Hz) stimulation and 80 s recovery phase. For each measurement, one arteriolar and one venular vessel segment of the superior temporal quadrant, one to two optic disc diameters away from the optic disc edge, were marked. Diameters of these segments were continuously recorded over time. Identical vessel segments were marked at both time points. The integrated RVA software (v.5.51; IMEDOS Systems GmbH, Jena, Germany) measured retinal microvascular function non-invasively by analyzing aFID and vFID based on the principles of neurovascular coupling (Garhofer et al., 2010). We have recently published standard operating procedures to describe how DVA should be conducted and how aFID and vFID should be analyzed (Streese et al., 2021a). These procedures were used to quantify aFID and vFID. Additionally, we investigated potential differences between our analytic approach and the manufacturer’s default method. For clarity, aFID and vFID based on the manufacture’s procedure will be described in this manuscript as aFID_mfr and vFID_mfr. Intraocular pressure was measured using a rebound tonometer (ICare PRO, Tiolat Oy, Helsinki, Finland).

### Statistical analysis

Statistical analyses and graphical visualization were performed using R version 3.6.1 or later (R Foundation for Statistical Computing, Vienna, Austria). Sample characteristics were descriptively described using mean and standard deviation or 95% confidence intervals (CI). After testing data for normal distribution, paired sample t-tests were calculated to analyze potential day-to-day differences (t1 vs. t2) of aFID and vFID for DVA 2.0 and DVA 3.0, as well as intra- and interobserver variability. We have used directed acyclic graphs (DAGs) to identify confounders and reduce risk of bias (Tennant et al., 2021). To assess intraobserver variability, a single experienced researcher reanalyzed 20 randomly selected signals after a 1-month interval. For interobserver variability, two experienced researchers independently analyzed 20 randomly selected signals, with aFID and vFID values compared between raters. In addition, we calculated the maximum arteriolar dilatation (aMAX) in µm based on the baseline diameter and aFID to account for potential variations in baseline arteriolar diameter. This parameter was calculated as: (baseline diameter/100) multiplied by (100 + aFID). Unpaired sample t-tests were calculated to analyze potential differences between the two DVA devices. The day-to-day variability was analyzed separately for DVA 2.0 and DVA 3.0 using the intraclass correlation coefficients (ICC). The ICCs were analyzed in R (R Foundation for Statistical Computing, Vienna, Austria) using the ICC () function from the psych (Revelle, 2024) package and interpreted based on Koo and Li (Koo and Li, 2016). Bland-Altman plots and scatterplots were used for visual interpretation of variability. Interdevice variability between DVA 2.0 and DVA 3.0 was analyzed based on the combined data from both time points. In addition, we analyzed the variability and potential differences between the manufactures defined method to analyze aFID and vFID and the recommended standard operating procedures previously defined by our working group (Streese et al., 2021a). All statistical tests were performed two-sided with a significance level of 0.05.

### Sample size calculation

The main outcome of this study was the ICC of the aFID and vFID, which was used to assess the day-to-day variability of the DVA 2.0 and DVA 3.0 systems. Twenty-six participants were required to reach a power of 80% with a significance level of 0.05, assuming a null hypothesis ICC0 of 0.60 and an anticipated ICC of 0.85 (Borg et al., 2022). To account for potential drop-outs or insufficient data quality, a drop-out rate of 20% was assumed that we finally invited 31 participants.

## Results

Thirty-one participants were invited to take part in this study. Three participants did not participate at both time points. The DVA signals of two participants had to be excluded due to poor quality. The final analysis included 26 participants (12 females), with a mean age of 43 ± 14 years. All participants were non-smokers. The mean baseline blood pressure (BP) of 134 ± 15 mmHg systolic and 86 ± 7 mmHg diastolic, while follow-up values were 131 ± 17 mmHg systolic and 83 ± 7 mmHg diastolic. Mean IOP was 16 ± 3 mmHg at baseline and 16 ± 2 mmHg at follow-up.

### Day-to-day variability

**FIGURE 1:**
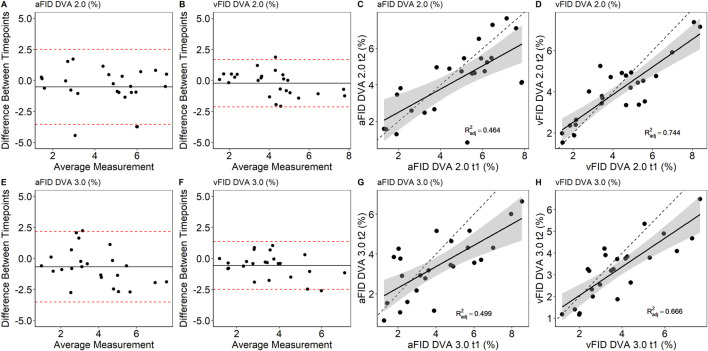
Bland-Altman plots and scatterplots to describe the day-to-day variability of aFID and vFID for DVA 2.0 **(A–D)** and DVA 3.0 **(E–H)**. aFID, arteriolar flicker light-induced dilation in %; vFID, venular flicker-light induced dilation in %; dotted line, *R* ^2^ = 1.

There were no statistically significant differences between aFID (t1: 4.8% ± 2.0% vs. t2: 4.3% ± 1.9%, p = 0.330) and vFID (t1: 4.2% ± 2.0% vs. t2: 4.0% ± 1.5%, p = 0.803) for DVA 2.0 and aFID (t1: 4.1% ± 2.1% vs. t2: 3.4% ± 1.5%, p = 0.426) and vFID (t1: 3.8% ± 1.7% vs. t2: 3.3% ± 1.4%, p = 0.392) for DVA 3.0. The ICCs for aFID and vFID ranged from 0.79 to 0.91 across both devices (Table 1). Bland-Altman plots and scatterplots for aFID and vFID of the day-to-day variability of both devices are shown in Figure 1.

**TABLE 1:** Mean, standard deviation and interclass correlation coefficients of the day-to-day variability of DVA 2.0 and DVA 3.0.

	DVA 2.0		DVA 3.0	
	aFID	vFID	aFID	vFID
ICC (CI)	0.81 (0.57, 0.92), p = 0.037	0.91 (0.80, 0.96), p < 0.001	0.79 (0.49, 0.91), p = 0.076	0.87 (0.61, 0.95), p = 0.022
	aFID_mfr	vFID_mfr	aFID_mfr	vFID_mfr
ICC (CI)	0.80 (0.55, 0.91), p = 0.048	0.89 (0.75, 0.95), p = 0.001	0.81 (0.56, 0.92), p = 0.041	0.88 (0.63, 0.95), p = 0.019

ICC, interclass correlation coefficient; CI, 95% confidence Interval; aFID, arteriolar flicker-light induced dilation; vFID, venular flicker-light induced dilation; aFID_mfr, aFID, based on the manufacture´s procedure; vFID_mfr, vFID, based on the manufacture´s procedure.

### Intra- and interobserver variability

**FIGURE 2:**
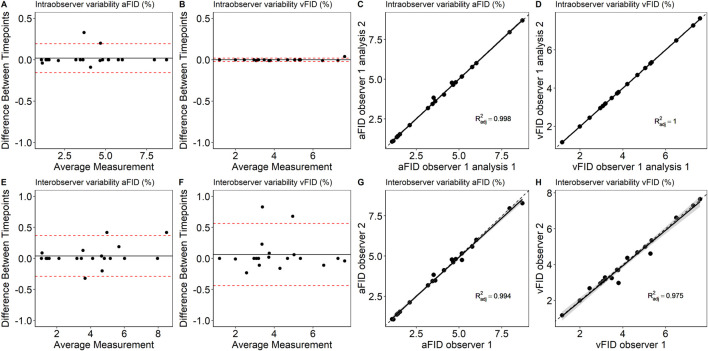
Bland-Altman plots and scatterplots to describe intra- **(A–D)** and interobserver variability **(E–H)** of aFID and vFID. aFID, arteriolar flicker light-induced dilation in %; vFID, venular flicker-light induced dilation in %; dotted line, *R* ^2^ = 1.

To assess intraobserver variability, a single experienced researcher reanalyzed 20 randomly selected DVA signals after a 1-month interval. The ICC for aFID and vFID was 0.999 (0.998, 1), p < 0.001. Interobserver variability was evaluated by two experienced researchers, each independently analyzing 20 randomly selected signals. The ICC for aFID and vFID was 0.997 (0.993, 0.999), p < 0.001 and 0.998 (0.971, 0.995), p < 0.001. Figure 2 shows the Bland-Altman plots and scatterplots for intra- and interobserver variability.

### Variability of DVA 2.0 and DVA 3.0

**FIGURE 3:**
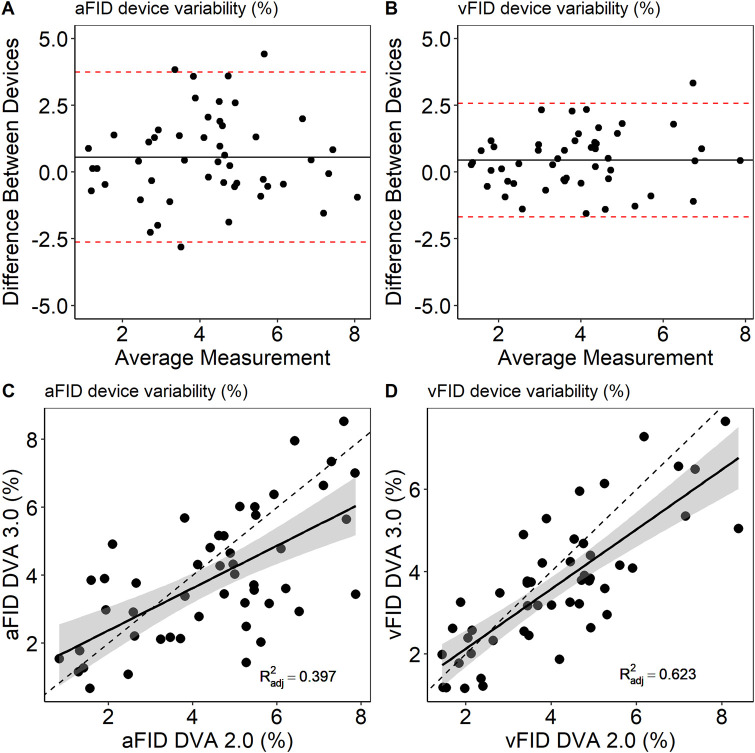
Bland-Altmann plots **(A,B)** and scatterplots **(C,D)** to describe interdevice variability of DVA 2.0 and DVA 3.0. aFID, arteriolar flicker light-induced dilation in %; vFID, venular flicker-light induced dilation in %; dotted line, *R* ^2^ = 1.

Measurements from both time points were pooled to analyze potential systematic differences between DVA 2.0 and DVA 3.0. There was no sign for systematic differences between DVA 2.0 and DVA 3.0. aFID (DVA 2.0: 4.6% ± 2.0% vs. DVA 3.0: 3.9% ± 1.9%, p = 0.073) and vFID (DVA 2.0: 4.1% ± 1.7% vs. DVA 3.0: 3.6% ± 1.6%, p = 0.143) showed no statistically significant differences. The ICC for the interdevice variability was 0.76 (0.57, 0.89), p = 0.042 for aFID and 0.87 (0.74, 0.93), p < 0.001 for vFID. Figure 3 shows the Bland-Altman plots and scatterplots for the aFID and vFID interdevice variability.

### Baseline variation

**FIGURE 4:**
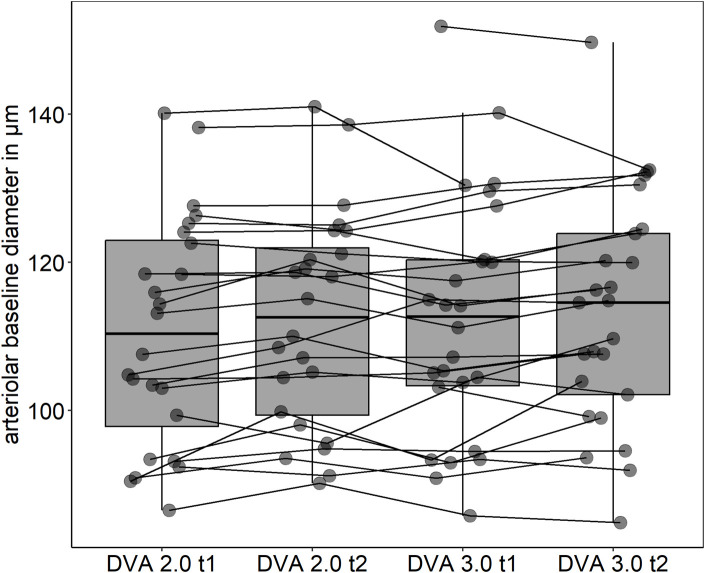
Individual arteriolar baseline variations. t1, timepoint one; t2, timepoint two.

**FIGURE 5:**
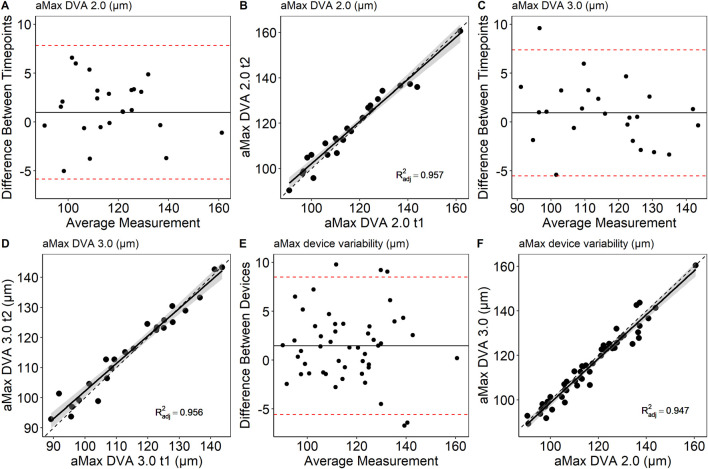
Bland-Altman plots and scatterplots to describe day-to-day variability **(A–D)** and interdevice variability **(E,F)** of aMAX. aMAX, maximum arteriolar widening in µm; dotted line, *R* ^2^ = 1.

Mean arteriolar baseline diameter of DVA 2.0 (112 ± 16 μm, 95% CI [106, 119] vs. 113 ± 16 μm, 95% CI [107, 120], p = 0.862) and DVA 3.0 (111 ± 15 μm, 95% CI [104, 117] vs. 112 ± 15 μm, 95% CI [106, 118], p = 0.714) were not statistically significant different between time points. Baseline arteriolar diameters were also comparable between devices at time point one (p = 0.685) and two (p = 0.813). However, individual variations were observed between devices and time points (Figure 4). To account for this variability, we calculated aMAX, which corrects aFID based on the corresponding bassline diameter. The day-to-day variability was lower for DVA 2.0 (0.988 (0.974, 0.995), p < 0.001) and DVA 3.0 (0.988 (0.972, 0.995), p < 0.001) after correcting for the baseline diameter by using the aMAX parameter (Figures 5A–D). Interdevice variability also decreased after correcting for baseline diameter variations by using aMAX (0.985 (0.965, 0.992), p < 0.001) (Figures 5E,F).

## Discussion

The day-to-day variability of both DVA 2.0 and DVA 3.0 ranged from poor to good according to the criteria suggested by Koo and Li (2016). More importantly, the day-to-day variability may be significantly reduced if baseline correction is applied. The intra- and interobserver variability was excellent for all assessed parameters, indicating high reproducibility using standardized procedures. However, the variability improved substantially following baseline diameter correction using the aMAX parameter. After correction, the ICC was excellent, according to Koo and Li (2016).

The retinal microcirculation is a valid vascular bed to quantify systemic CV risk (Hanssen et al., 2022). DVA is a unique and non-invasive method to quantify microvascular function as a biomarker for systemic CV risk. In our study, the day-to-day variability ranged from poor to good. aFID showed moderate ICCs for DVA 2.0 and poor ICCs without statistical significance for DVA 3.0. A previous study showed higher levels of reproducibility (Pache et al., 2002). Interestingly the ICCs varied depending on whether aFID and vFID values were generated manually using previously defined standard operating procedures (Streese et al., 2021a) or the manufacture´s default procedure. For DVA 2.0 aFID and vFID, analyzed with standard operating procedures, showed higher ICCs compared to manufacture´s procedure. For DVA 3.0 aFID and vFID generated by manufacture´s procedures showed higher ICCs compared to manually assessed values based on the standard operating procedures. However, the intra- and interobserver variability was excellent for aFID and vFID assessed with the standard operating procedures in our study. Analyses from different examiners seem to be comparable when our previous defined standard operating procedures (Streese et al., 2021a) are used. Our results represent very important and necessary steps towards clinical implementation. Nevertheless, further technical improvements such as automated analysis and interpretation of signals are likely to achieve comparable high levels of operator independency in less trained individuals as well as higher levels of reproducibility.

Intraindividual day-to-day variations may thus limit the potential to use this technique to monitor individual disease progression. In our EXAMINE AGE study, we have recently demonstrated that healthy and very active individuals showed similar aFID compared to CV risk patients (Streese et al., 2019). This phenomenon could be attributed to the differences in arteriolar diameters between the two groups prior to the flicker light stimulation. When the arteriolar diameter was reduced by BP-induced myogenic constriction before flicker light stimulation, a significant better response was observed in the healthy and active group compared to the CV risk patients (Streese et al., 2021b). We have previously shown that the individual baseline diameter is an important driver for the arteriolar flicker-light response (Streese et al., 2019; Streese et al., 2021b). Although we did not observe a significant difference in baseline diameters between the two time points of arterioles and venules in our sample, the values exhibit considerable variation. With comparable maximum absolute diameters after flicker light stimulation these variations in baseline diameter markedly influence the percentage-based dilatory capacity. Similar issues have already been reported with other percentage-based methods (Atkinson and Batterham, 2013). Therefore, the resting diameter should always be considered. The present study, along with the improvements in day-to-day variability after correcting for arteriolar baseline diameter (aMAX), assuming Gullstrand’s normal eye, underscores the importance of considering both vessel structure and functional capacity when interpreting microvascular health. We therefore recommend to correct for baseline diameter variations when monitoring disease progression in individuals.

Several physiological factors might be responsible for the baseline diameter variability, such as alterations of blood pressure, heart rate or breathing frequency. To minimize these influencing factors, we highly recommend to standardize the time of the day as well as nicotine, nutrition, alcohol and exercise prior to the examination. The baseline diameter variation can also be a consequence of different brightness during the DVA. Even before the flicker-light starts the eye is exposed to bright light, which varies day by day based on the patient’s form of the day.

Our analyses showed no evidence for a systemic difference between DVA 2.0 and DVA 3.0. However, the mean device difference was 0.56% and 0.44% for aFID and vFID, with some individual differences of more than 3.7% or 2.6% for aFID and vFID. Such high variations might lead to misinterpretation of individual CV risk profile. It appears eminent to use the same device to analyze individual disease progression longitudinally. The upside is, these interdevice differences were massively reduced after correction for baseline variations. Therefore, it seems highly important to correct for individual baseline variations whenever repeated measurements are planned. Based on the uncritical interdevice variability, after baseline correction, we assume that the recent published normative data (Streese et al., 2021a), expended with normative values for the maximal diameter (Supplementary Figures S1–3) generated with DVA 2.0, can also be used to interpret the individual CV risk of patients when data were generated with the DVA 3.0. Whether our results are producible in clinical settings remains to be elucidated in future studies. The baseline variation in patients may be different to our healthy cohort. We recommend to control for arteriolar baseline variations as this might mask potential group differences, risk associations or intervention effects in clinical settings. By considering baseline diameter we may be able to further increase precision measurement using dynamic retinal vessel analysis.

Our study has several limitations. First, the small sample size may not be sufficient to detect systematic differences between the two devices. Missing sample characteristics such as body mass index or physical activity levels of participants might limit the generalizability of the results. The recruiting procedure may have resulted in a higher sample heterogeneity. This is a proof of principle approach and future research will have to invest the generalizability of the method in different and less heterogenic populations and diseases. Furthermore, at this stage, it is not possible to distinguish whether the variability in baseline diameters arises from biological factors, differences in the technical properties of the devices, or a combination of both. The correction for baseline diameter variations by calculating aMAX and thus combining absolute diameter values with the relative dilation capacity, necessitates meticulous standardization of examination protocols and image analyses. Future studies in clinical settings need to confirm the advantage of this new parameter.

In conclusion, DVA appears to be a robust method to quantify retinal microvascular function independent of device generation. Standard percentage-based metrics are appropriate for risk stratification at an individual level and at single time point. However, for longitudinal monitoring, or interdevice comparison within the same cohort, we recommend incorporating baseline diameter correction to reduce variability. The intra- and interobserver variability is neglectable if standard operating procedures are adhered to. However, future studies need to consider the variation of the arteriolar baseline diameters to achieve highest levels of reproducibility and data quality. Future methodological studies need to address differences between biological and technical factors which may contribute to variation of arteriolar or venular flicker-light responses.

## Data Availability

The raw data supporting the conclusions of this article will be made available by the authors, without undue reservation.
