# Supplementary material for: Device-based day-to-day and observer variability to quantify dilation capacity in the retinal microcirculation
Source: Front Physiol. 2025 Oct 15;16:1663370. doi: 10.3389/fphys.2025.1663370 (PMC12568701; doi:10.3389/fphys.2025.1663370)
Supplement: Supplementary file 1 [file Presentation1.pdf]

Supplementary Material

Device-based day-to-day and observer variability to quantify dilation capacity in the retinal microcirculation

Lukas Streese[1,2†], Christoph Hauser[1†], Denis Infanger[1], Sascha Klee[3,4], Dietmar Link[4], Walthard Vilser[4], Henner Hanssen[1*]

[1]Department of Sport, Exercise and Health, Medical Faculty, University of Basel, Basel, Switzerland

[2]Faculty of Health Care, Niederrhein University of Applied Sciences, Krefeld, Germany

[3] Division Biostatistics and Data Science, Department General Health Studies, Karl Landsteiner University of Health Sciences, Krems an der Donau, Austria

[4]Division Optoelectrophysiological Engineering, Department of Computer Science and Automation, Institute of Biomedical Engineering and Informatics, Technische Universität Ilmenau, Ilmenau, Germany

***Address for Correspondence**

Prof Henner Hanssen, MD

University of Basel

Medical Faculty

Department of Sport, Exercise and Health

4052 Basel, Switzerland

Tel: +41 61 207 47 46

email: henner.hanssen@unibas.ch

Supplement Figure 1. Normative data for aMAX of both sexes.

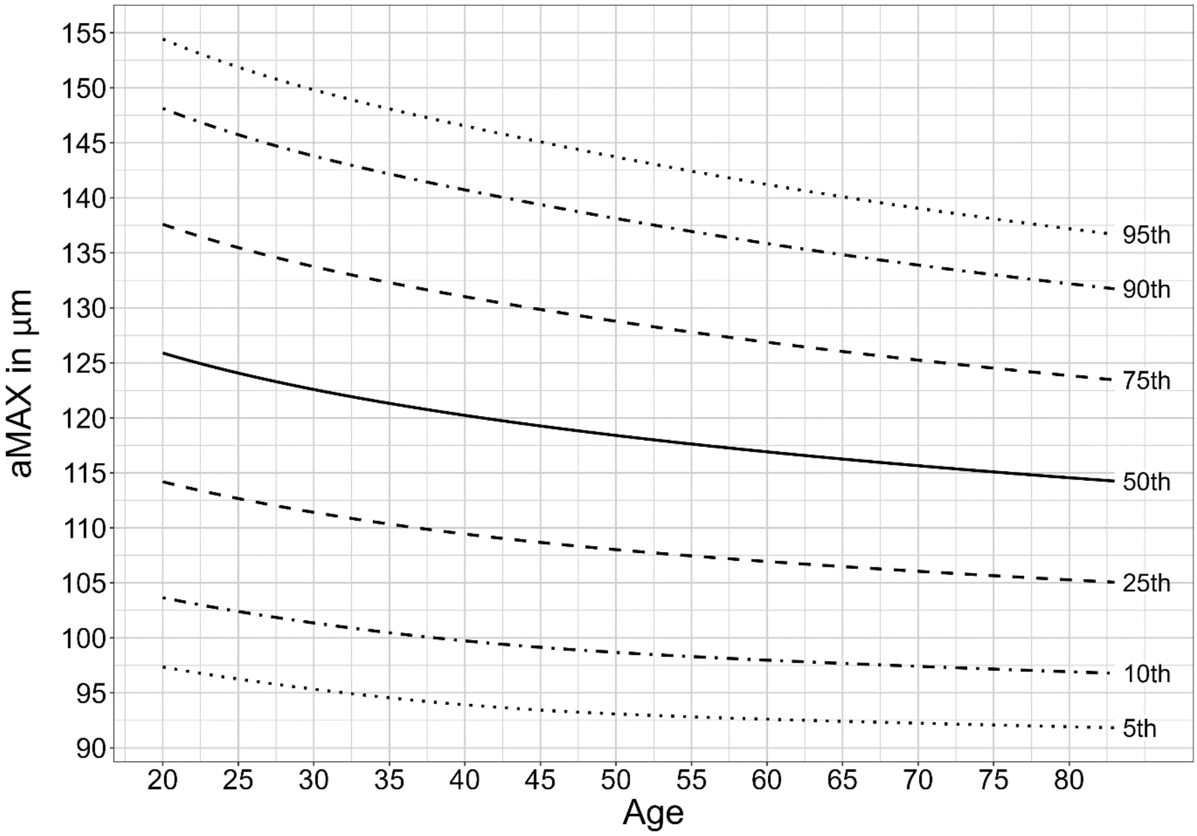

Normative data for maximum arteriolar dilatation (aMAX) in µm of 267 females and males from the COMPLETE-HEALTH study[1].

Supplement Figure 2. Normative data for aMAX of females.

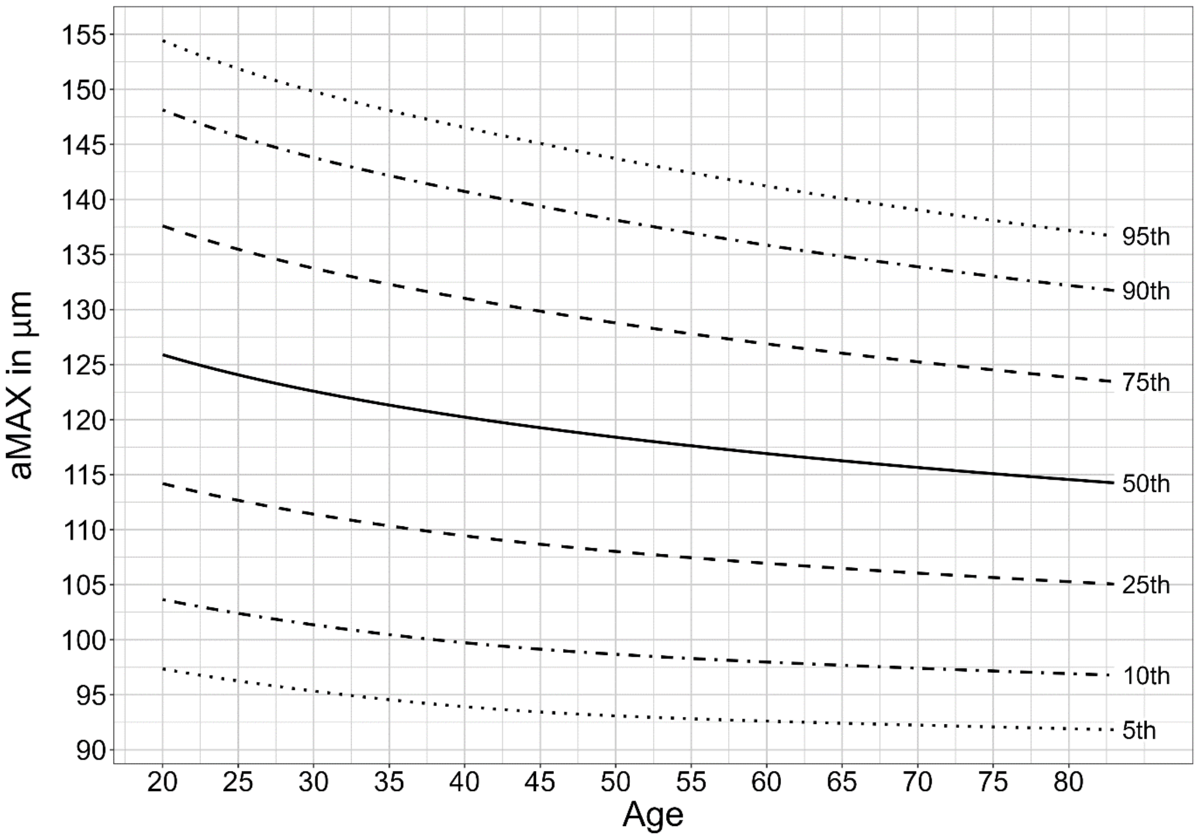

Normative data for maximum arteriolar dilatation (aMAX) in µm of 123 females from the COMPLETE-HEALTH study[1].

Supplement Figure 3. Normative data for aMAX of males.

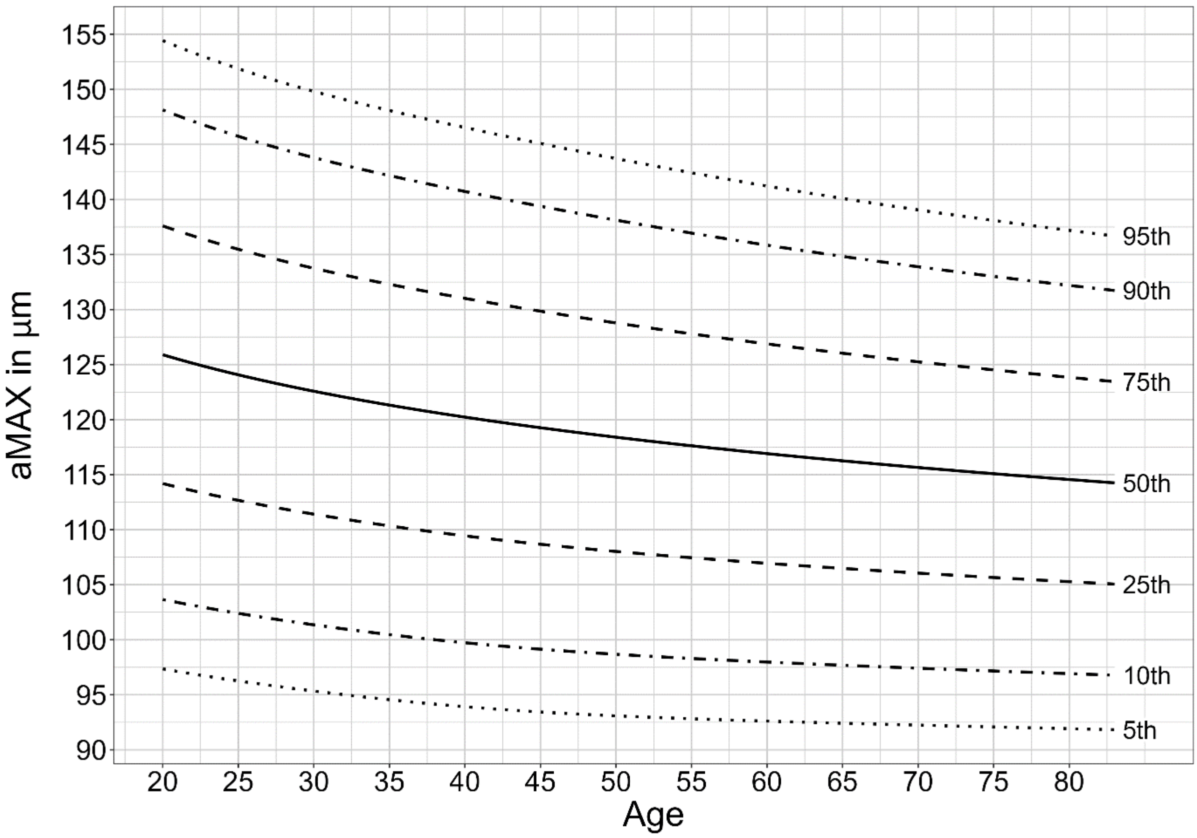

Normative data for maximum arteriolar dilatation (aMAX) in µm of 144 males from the COMPLETE-HEALTH study[1].

References

1. Streese L, Lona G, Wagner J, Knaier R, Burri A, Neve G, Infanger D, Vilser W, Schmidt-Trucksass A, Hanssen H. Normative data and standard operating procedures for static and dynamic retinal vessel analysis as biomarker for cardiovascular risk. *Sci Rep* 2021;**11**:14136.
